# Determination of Axial Length Requiring Adjustment of Measured Circumpapillary Retinal Nerve Fiber Layer Thickness for Ocular Magnification

**DOI:** 10.1371/journal.pone.0107553

**Published:** 2014-09-12

**Authors:** Kazunori Hirasawa, Nobuyuki Shoji, Yukako Yoshii, Shota Haraguchi

**Affiliations:** Orthoptics and Visual Science, Department of Rehabilitation, School of Allied Health Sciences, Kitasato University, Kanagawa, Japan; Medical University Graz, Austria

## Abstract

**Purpose:**

To determine the axial length requiring adjustment of measured circumpapillary retinal nerve fiber layer (cpRNFL) thickness to account for ocular magnification during spectral-domain optical coherence tomography (SD-OCT).

**Methods:**

In this prospective study, 148 eyes of 148 healthy student volunteers were imaged by two examiners using three-dimensional SD-OCT. In 54 randomly selected eyes, total cpRNFL thickness was measured with and without adjustment for ocular magnification to establish intra-examiner and inter-examiner measurement error. The 148 eyes were then divided into three groups according to the error values: control group (difference in the corrected and uncorrected total cpRNFL thickness was within the measurement error range), thinner group (the corrected total cpRNFL thickness was less than the uncorrected one), and thicker group (the corrected total cpRNFL thickness was more than the uncorrected one). The cutoff values of axial length between the control and the other groups were calculated by receiver operating characteristic analysis.

**Results:**

Measurement error ranged from 4.2 to 5.3 µm; the threshold value was defined as 5.3 µm. The cutoff values of axial length between the thinner and the control groups and between the control and the thicker groups were 23.60 (area under the curve [AUC] = 0.959) and 25.55 (AUC = 0.944) mm, respectively.

**Conclusions:**

Axial lengths shorter than 23.60 mm and longer than 25.55 mm require adjustment of measured cpRNFL thickness to account for ocular magnification during SD-OCT.

**Clinical Trial Registration:**

UMIN Clinical Trials Registry (http://www.umin.ac.jp/) under unique trial number UMIN000013248 (date of registration: 02/24/2014)

## Introduction

Circumpapillary retinal nerve fiber layer (cpRNFL) thickness measured by optical coherence tomography (OCT) is extensively used for glaucoma diagnosis. [1]–[5] It is generally measured in an imaged scan circle approximately 3.4 mm in diameter and centered on the optic nerve head based on the Gullstrand schematic eye (corneal radius = 7.7 mm; refraction = 0 diopter; axial length = 24.39 mm). As the apparent size of the optic nerve head is magnified in hyperopic eyes (short axial length) and minified in myopic eyes (long axial length), [6]. the scan circle must be enlarged and reduced, respectively. Accordingly, measured cpRNFL thickness should also be corrected to account for ocular magnification, a drawback of the imaging device.

Reportedly, uncorrected cpRNFL thickness has a negative correlation with axial length. [7]–[18] On the other hand, corrected cpRNFL thickness has no correlation or only a weak positive correlation with axial length [15]–[18].

Myopia is highly prevalent worldwide. [19]–[24] Myopia and hyperopia are risk factors for open-angle [25]–[29] and angle-closure [30]–[32] glaucoma, respectively, implying that these defects are inseparable from glaucoma. Of note, the axial lengths requiring correction of measured cpRNFL thickness to account for ocular magnification have not been evaluated. The aim of this study was to determine the axial lengths requiring adjustment of cpRNFL thickness measurements for ocular magnification during spectral-domain OCT (SD-OCT).

## Methods

### Participants

One hundred forty-eight student volunteers from Kitasato University were recruited. The study followed the tenets of the Declaration of Helsinki, and written informed consent was obtained from each participant after receiving approval from the Ethics Committee of Kitasato University School of Allied Health Science (No. 2012-07).

All the volunteers underwent comprehensive ophthalmic examinations including noncycloplegic refraction testing (KR-8100PA, Topcon, Japan), visual acuity testing at 5 m using a Landolt ring chart, intraocular pressure (NT-530P, NIDEK, Japan) and axial length measurements (OA-1000, TOMEY, Japan), and fundus examination by a glaucoma specialist. Those with corrected visual acuity of 20/20 or better, intraocular pressure of 21 mmHg or less, normal optic disc, and no fundus disease were included.

### SD-OCT

SD-OCT (3D OCT-2000, version 8.00; Topcon, Japan) was used for cpRNFL thickness measurement. The device operates at a speed of 50, 000 A-scans per second and has a depth and lateral resolution of 6 and 20 µm or less, respectively. It requires a pupil size of 2.5 mm or larger for imaging. The measurements were performed in three-dimensional optic disc scan mode, consisting of 512 A-scans per B-scan×128 C-scan resolution and a 6×6 mm scan area.

Uncorrected total cpRNFL thickness was measured in a 3.4 mm diameter scan circle centered on the optic nerve head based on the Gullstrand schematic eye. The imaging device automatically adjusts the scan circle diameter to account for ocular magnification on the basis of both Littman’s method [33] and Littman’s method modified by Bennett et al. [6] after determining refraction, corneal curvature, and axial length.

### Quantification of measurement error and grouping

One randomly selected eye of each participant was imaged with and without correction, in random order, by either examiner A or examiner B. Mydriatic agent were not used. The examiners imaged an equal number of eyes. Total cpRNFL thickness of 54 randomly selected eyes was measured by both examiners to quantify measurement error. For inter-examiner measurement error, the second measurements were analyzed.

The obtained measurement error values were used to categorize the all eyes as follows:

- Control group: difference in the corrected and uncorrected total cpRNFL thickness was within the measurement error range;
- Thinner group: the corrected total cpRNFL thickness was less than the uncorrected one; and
- Thicker group: the corrected total cpRNFL thickness was greater than the uncorrected one.

Images with a quality of 70 or less and lack or deviation of OCT line images after examination were excluded.

### Statistical analysis

All the data were compiled into a Microsoft Excel worksheet and analyzed by using statistical software (SPSS version 19.0, IBM Japan, Ltd., Tokyo, Japan; MedCalc version.12.3.0, MedCalc Software, Ostend, Belgium; and G*Power 3 version 3.1.7, Faul F, Universität Kiel, Germany).

Associations between axial length and cpRNFL thickness with and without adjustment for ocular magnification were analyzed by using Pearson product-moment correlation. The effect size, α error, and power (1-β error) were determined to be 0.30, 0.05, and 0.90, respectively, by a two-tailed test and the required sample size was 109 eyes.

Measurement error was calculated according to the Bland and Altman method as 2.77*S_w_, [34], [35] where S_w_, the within-subject standard deviation, is the square root of the average of within-subject variances. When the number of measurements and confidence interval on either side of the estimate of S_w_ were set to 2 and 0.20, respectively, the required sample size was 44 eyes [36].

The cutoff values of axial length requiring adjustment of measured cpRNFL thickness were derived from the maximum value of the Youden Index [37] and calculated by receiver operating curve (ROC) analysis. When type I error (α error) was 0.01, type II error (β error) was 0.01, and null hypothesis value of the area under the curve (AUC) was 0.50 and the expected AUC values were set to 0.80, 0.85, 0.90, and 0.95, the required sample sizes for this analysis were 44, 31, 23, and 17 eyes, respectively.

## Results

Sixteen participants (six and 10 participants measured by examiners A and B, respectively) were excluded because they were less than images quality of 70 or less and lack or deviation of OCT line images after examination, so 132 eyes of 132 participants (68 and 64 participants measured by examiners A and B, respectively) were analyzed. Their demographic data are shown in Table 1.

**Table 1:** Demographic and Ocular Characteristics of the Participants.

Parameter	Mean ± SD	Range
Participants (men/women)	132 (41/91)	
Assessed eye (right/left)	132 (65/67)	
Age (years)	21.7±1.6	20 to 28
Spherical refraction (diopters)	−3.02±3.08	−13.00 to 4.00
Astigmatism (diopters)	−0.68±0.83	−6.00 to 0.00
Corneal radius (mm)	7.79±0.27	6.93 to 8.42
Visual acuity (logMAR)	−0.23±0.07	−0.30 to 0.00
Axial length (mm)	24.78±1.51	21.25 to 28.35
Corneal thickness (µm)	539.3±24.5	472.0 to 595.0
Intraocular pressure (mmHg)	14.4±2.2	7.7 to 20.0

SD = standard deviation; logMAR = logarithm of the minimum angle resolution.

**Figure 1:**
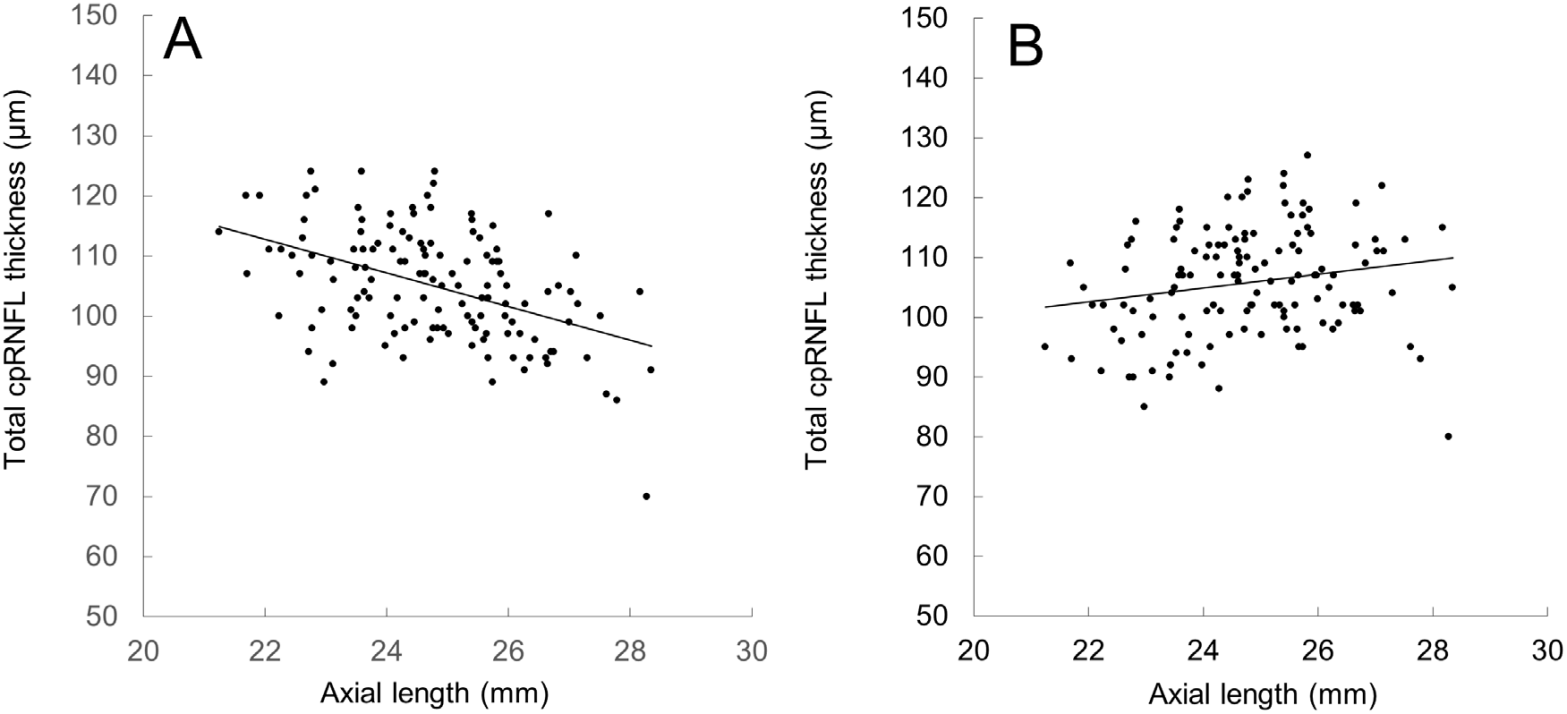
Correlation of axial length and total cpRNFL thickness measured with and without adjustment for ocular magnification. (A) Uncorrected total cpRNFL thickness decreased as axial length increased (y = −2.8015x+174.45; R^2^ = 0.208; p<0.001) whereas (B) the corrected one increased as axial length increased (y = 1.1597x+77.068; R^2^ = 0.039; p = 0.024).

As shown in Figure 1, uncorrected and corrected total cpRNFL thickness showed negative and weak positive correlations with axial length, respectively. For every 1 mm increase in axial length, uncorrected total cpRNFL thickness decreased by 2.8 µm (*p*<0.001) and the corrected one slightly increased by 1.2 µm (*p* = 0.024).

With regard to measurement error, three of the 54 participants were excluded because they were less than images quality of 70. Therefore, 51 eyes of 51 participants were analyzed. The measurement error values with and without adjustment ranged from 4.4 to 5.3 µm (Table 2). The threshold value of measurement error was set as 5.3 µm. Accordingly, 72, 25, and 35 eyes were classified into the control, thinner, and thicker groups, respectively.

**Table 2:** Measurement Error Values of Total cpRNFL Thickness.

Adjusted for ocularmagnification	Examiner A (µm)	Examiner B (µm)	Inter-examiner (µm)
Yes	5.2	4.4	5.3
No	4.9	4.8	4.7

cpRNFL = circumpapillary retinal nerve fiber layer; S_w_ = within-subject variation.

The values were calculated as 2.77*S_w_.

The cutoff values of axial length between the thinner and the control groups was 23.60 mm (AUC = 0.959) and between the control and the thicker groups was 25.55 mm (AUC = 0.944). The data of the ROC analysis are shown in Table 3.

**Table 3:** Results of the ROC Analysis.

Comparison	Axial lengthcutoff (mm)	AUC	Sensitivity	Specificity	Youden Index
Thinner vs. control group	23.60	0.959	0.889	0.809	0.698
Control vs. thicker group	25.55	0.944	0.943	0.790	0.733

ROC = receiver operating characteristic; AUC = area under the curve.

## Discussion

In this study, uncorrected total cpRNFL thickness was negatively correlated with axial length and corrected total cpRNFL thickness showed a weak positive correlation with axial length. The variation in uncorrected total cpRNFL thickness per millimeter of axial length would not be negligible. This finding implies that adjustment for ocular magnification is important to measure cpRNFL thickness accurately.

Previous studies showed that uncorrected cpRNFL thickness decreased in the range of −1.8 to −4.8 µm as axial length increased. [7]–[9], [11], [13], [14], [17] On the other hand, corrected cpRNFL thickness increased by 2.2 µm with increasing axial length. [17] When cpRNFL is analyzed at 3.4 mm diameter from the center of the optic disc without correction, the fundus image is minified in the case of longer axial length and the cpRNFL is assessed distantly to the optic disc, whereas that for shorter axial length is magnified and the cpRNFL is analyzed nearer to the optic disc. The actual scan circle diameter adjusted for magnification will be closer to the optic disc in myopic eyes and distant to the optic disc in hyperopic eyes, and the measured cpRNFL will be thicker and thinner, respectively. However, the variation in cpRNFL thickness is not really linear because the cpRNFL is distributed radially. cpRNFL thickness increases nearer to the optic disc but the variation is small compared with the peripheral region. [37] Therefore, the variation in cpRNFL thickness will be greater in the case of long axial length than short axial length, suggesting a weak positive correlation between cpRNFL thickness and axial length.

The measurement error values with and without adjustment ranged from 4.4 to 5.3 µm. Intra-examiner and inter-examiner measurement error values by SD-OCT are reportedly in the range of 3.1 to 11.7 µm. [38]–[52] The lower values in the present study can be attributed to good fixation during imaging because the participants were young. They also suggest lack of operator bias with the imaging technique. Therefore, 5.3 µm would be a valid criterion to categorize patients.

With the threshold measurement error value of 5.3 µm, the cutoff values of axial length requiring adjustment of measured cpRNFL thickness for ocular magnification were 23.60 and 25.55 mm. cpRNFL thickness was measured in total instead of in quadrant and clock-hour sectors because measurement in smaller sectors reduces repeatability gradually. [38]–[52] Further, structural changes of the fundus should be considered: in the so-called “temporal shift,” in which the superotemporal and inferotemporal arteries and veins are more closely located toward the temporal horizon in eyes with increased axial length, the peak of the double hump of cpRNFL thickness also shifts toward the temporal horizon. [10], [13] Importantly, if a measurement error value smaller than 5.3 µm is used, the cutoff range of axial length narrows, and vice versa. Therefore, 23.60 and 25.55 mm should be recognized as reference values rather than absolute values.

The calculated cutoff values of axial length represent moderate hyperopia and myopia. Because of the recent high prevalence of myopia, [19]–[24] the number of people with longer axial lengths of 25.55 mm is expected to increase worldwide. Therefore, cpRNFL thickness would require careful assessment in myopia, especially in a cross-sectional sample. If cpRNFL thickness of hyperopic or myopic eyes is imaged without adjustment, it will be overestimated or underestimated, respectively. On the other hand, in a longitudinal evaluation of the same condition, the influence of ocular magnification would be negligible. However, when mixed-result with and without adjustment was evaluated intra-participants, it is needed for careful to misreading depending on the axial length.

In conclusion, measured cpRNFL thickness is significantly influenced by whether or not it is corrected for ocular magnification depending on axial length. If the measurement error of cpRNFL thickness is 5.3 µm, the influence of ocular magnification might be small for axial lengths in the 23.60–25.55 mm range. However, in eyes with axial lengths shorter than 23.60 mm or longer than 25.55 mm, cpRNFL thickness can be accurately measured and compared among subjects if it is corrected for ocular magnification.
